# Effects of gravity changes on gene expression of BDNF and serotonin receptors in the mouse brain

**DOI:** 10.1371/journal.pone.0177833

**Published:** 2017-06-07

**Authors:** Chihiro Ishikawa, Haiyan Li, Rin Ogura, Yuko Yoshimura, Takashi Kudo, Masaki Shirakawa, Dai Shiba, Satoru Takahashi, Hironobu Morita, Takashi Shiga

**Affiliations:** 1 Laboratory of Neurobiology, Graduate School of Comprehensive Human Sciences, University of Tsukuba, Tsukuba, Japan; 2 Laboratory Animal Resource Center, University of Tsukuba, Tsukuba, Ibaraki, Japan; 3 Department of Anatomy and Embryology, Faculty of Medicine, University of Tsukuba, Tsukuba, Japan; 4 Mouse Epigenetics Project, ISS/Kibo experiment, Japan Aerospace Exploration Agency (JAXA), Tsukuba, Japan; 5 JEM Utilization Center, Human Spaceflight Technology Directorate, JAXA, Tsukuba, Ibaraki, Japan; 6 Department of Physiology, Gifu University Graduate School of Medicine, Gifu, Japan; 7 Department of Neurobiology, Faculty of Medicine, University of Tsukuba, Tsukuba, Ibaraki, Japan; Kent State University, UNITED STATES

## Abstract

Spaceflight entails various stressful environmental factors including microgravity. The effects of gravity changes have been studied extensively on skeletal, muscular, cardiovascular, immune and vestibular systems, but those on the nervous system are not well studied. The alteration of gravity in ground-based animal experiments is one of the approaches taken to address this issue. Here we investigated the effects of centrifugation-induced gravity changes on gene expression of brain-derived neurotrophic factor (BDNF) and serotonin receptors (5-HTRs) in the mouse brain. Exposure to 2*g* hypergravity for 14 days showed differential modulation of gene expression depending on regions of the brain. BDNF expression was decreased in the ventral hippocampus and hypothalamus, whereas increased in the cerebellum. 5-HT1BR expression was decreased in the cerebellum, whereas increased in the ventral hippocampus and caudate putamen. In contrast, hypergravity did not affect gene expression of 5-HT1AR, 5-HT2AR, 5-HT2CR, 5-HT4R and 5-HT7R. In addition to hypergravity, decelerating gravity change from 2*g* hypergravity to 1*g* normal gravity affected gene expression of BDNF, 5-HT1AR, 5-HT1BR, and 5-HT2AR in various regions of the brain. We also examined involvement of the vestibular organ in the effects of hypergravity. Surgical lesions of the inner ear’s vestibular organ removed the effects induced by hypergravity on gene expression, which suggests that the effects of hypergravity are mediated through the vestibular organ. In summary, we showed that gravity changes induced differential modulation of gene expression of BDNF and 5-HTRs (5-HT1AR, 5-HT1BR and 5-HT2AR) in some brain regions. The modulation of gene expression may constitute molecular bases that underlie behavioral alteration induced by gravity changes.

## Introduction

Brain functions are modulated by various environmental factors including stress. The environmental factors entailed in spaceflight reportedly affect human physiological and psychological functions [1–4]. Life during spaceflight includes multiple stressful environmental factors, such as microgravity, radiation, loss of light-dark cycle, and confinement. Understanding the effects of these environmental factors is important for biological research on adaptation to environmental changes and for maintaining the health conditions of crewmembers during spaceflight.

Among these factors, the effects of gravity changes on skeletal, muscular, cardiovascular, immune and vestibular systems have so far been extensively studied using humans and experimental animals. In contrast, the effects of gravity changes on the nervous system are not fully understood, given the brain’s diverse structure and function. Animal experiments under both spaceflight and ground-based conditions have revealed that hypergravity as well as microgravity modulate rodent brain function and behavior, including cognition and emotion [5–10]. For example, hypergravity impairs learning ability [6,7,10], and also increases the anxiety level and stress response [5,9]. However, the molecular mechanisms underlying the effects of gravity changes on behavior have not been extensively investigated [11–15]. Recently, spaceflight experiments using the Russian biosatellite Bion M1 reported that one-month of spaceflight affected gene expression of several trophic factors and monoaminergic systems in selected mouse brain regions [16–18]. For example, the expression of glial cell line-derived neurotrophic factor was reduced in the striatum and hypothalamus, while increased in the frontal cortex by the spaceflight [18]. The expression of brain-derived neurotrophic factor (BDNF) was not affected in the same condition [16]. Among monoaminergic systems, the expression of serotonin 2A receptor (5-HT2AR) and dopamine D1 receptor was reduced in the hypothalamus [17]. However, there are still limited studies on spaceflight and ground-based experiments that investigated gene expression of functional molecules in the brain under altered gravity. Based on the previous studies reporting that BDNF and the 5-HT system are involved in various brain functions including anxiety, depression, aggression, learning and memory, and that dysregulations of these molecules are closely related to neuropsychiatric diseases [19–23], we hypothesized that these molecules are vulnerable to gravity changes and most likely mediate the effects of gravity changes on various types of behavior.

As the ground-based experiments of gravity changes, centrifugation and tail-suspension have been used as hypergravity and microgravity, respectively. However, tail-suspension produces microgravity only in the lower limbs, and is not suitable to examine the effects of gravity on brain function. Therefore, in the present study, in order to elucidate the molecular bases for the effects of gravity changes on behavior, we investigated the effects of centrifugation-induced hypergravity on gene expression of BDNF and 5-HTRs in various regions of the mouse brain.

## Materials and methods

### Mice

C57BL/6J male mice were purchased from Chubu Kagaku Shizai (Nagoya, Japan). The animals were handled in accordance with the “Guiding Principles for Care and Use of Animals in the Field of Physiological Science” prescribed by the Physiological Society of Japan. All the experiments in the present study were approved by the Animal Research Committees of Gifu University (Gifu, Japan) and University of Tsukuba (Tsukuba, Japan), and the Institutional Animal Care and Use Committee of Japan Aerospace Exploration Agency (JAXA).

### Induction of hypergravity

The 2*g* hypergravity in the dorsoventral direction was applied to mice in the prone posture by centrifugation using the gondola-type centrifuge (Shimazu, Kyoto, Japan) for 14 days [24,25]. Mice were kept in aluminum cages (35 x 25 x 17 cm) that were placed inside the rotating box of the centrifuge. After exposure to hypergravity, some mice were further kept for three days in the same cages under normal 1*g* gravity to examine recovery from hypergravity-induced changes. All the mice had access to food and water ad libitum, and room temperature was maintained at 24 ± 1°C with a 12:12 h light-dark cycle. During a daily 30 min of break, the cages were cleaned, with water and food being replenished. The control animals were placed near the centrifuge, but not exposed to hypergravity.

At the brain dissection after exposure to gravity changes, the weight of lower limb muscles were measured and the effects of hypergravity on the weight increase of soleus muscles, anti-gravity muscles, were confirmed (S1 Table).

### Brain dissection

Under deep anesthesia using isoflurane (Escain, Pfizer, Tokyo, Japan), mice were decapitated and their brains were removed quickly. Coronal slices at 2-mm thickness were made from the frontal pole of the cerebrum using Mouse Brain Matrix (Muromachi Kikai Co., Ltd), and the left hemisphere was used for the analysis of mRNA expression. The medial prefrontal cortex, amygdala, and dorsal raphe nucleus were punched out using the Harris Micro-Punch (GE healthcare) (S1 Fig). In addition, Noyes surgical scissors were used to dissect the caudate putamen (striatum), dorsal and ventral hippocampi, hypothalamus and cerebellar vermis (S1 Fig). Each brain region was quickly frozen in liquid nitrogen and stored at -80^°^C until use. We focused on these brain regions because these regions express BDNF and 5-HTRs abundantly, and are involved in various behaviors including learning/memory, anxiety, depression, or motor functions [19–21,23].

### Real-time reverse transcription-PCR

Real-time reverse transcription PCR was performed as previously described [26]. In brief, each brain region was homogenized in RNAiso (Takara) on ice using a sonicator (Taitec). Total RNA was isolated using RNAiso as per the manufacturer’s instructions. The isolated 1 μg of RNA was reverse-transcribed using the QuantiTect Reverse Transcription Kit (Qiagen). PCR was performed using the Thermal Cycler Dice Real Time System TP800 (Takara) with SYBR Premix Ex Taq II (Takara). Table 1 lists the primer sequences. Quantitation was performed using the crossing point method, and data was normalized to 18S rRNA. We confirmed that gravity changes did not have significant effects on the expression of 18S rRNA (S2 Table)

**Table 1:** Primer sequences.

gene	sequence	final conc. (nM)
18s	F: 5'-actcaacacgggaaacctc-3'	400
	R: 5'-aaccagacaaatcgctcca-3'	400
5-HT1AR	F: 5'-ccgtgagaggaagacagtgaaga-3'	200
	R: 5'-ggttgagcagggagttggagtag-3'	200
5-HT1BR	F: 5'-acatcctcggtcacctccatta-3'	400
	R: 5'-ccctagcggccatgagtttc-3'	400
5-HT2AR	F: 5'-ccagcggtccatccacagag-3'	200
	R: 5'-accacattacaacaaacagaaagaacac-3'	200
5-HT2CR	F: 5'-ggtccttcgtggcattcttcatc-3'	200
	R: 5'-cgcagttcctcctcggtgtg-3'	200
5-HT4R	F: 5'-gtatgaatggccagctgacaagaa-3'	400
	R: 5'-cccactggtaactacacatggacaa-3'	400
5-HT7R	F: 5'-ctgcagggtccttgtgactttc-3'	400
	R: 5'-tcaggagccttcaggagtgtg-3'	400
BDNF	F: 5'-gacaaggcaacttggcctac-3'	400
	R: 5'-actgtcacacacgctcagctc-3'	400
Tph2	F: 5'-gagcagggttactttcgtccatc-3'	400
	R: 5'-aagcaggtcgtctttgggtca-3'	400
TrkB	F: 5'-cattcactgtgagaggcaacc-3'	400
	R: 5'-atcagggtgtagtctccgttatt-3'	400

### Lesion of vestibular organ

The inner ear’s vestibular organ is a sense organ to detect gravity. To examine the role of vestibular organ in the modulation of gene expression under hypergravity, six-week-old mice were anesthetized by inhaling isoflurane via a face mask, and the vestibular organ was lesioned through an external auditory meatus using operation microscope (OMS-75, Topcon, Tokyo, Japan) as previously described [24,25,27]. After the removal of the tympanic membrane and the auditory ossicles, labyrinthine fluid was aspirated. Subsequently, #20 or #25 file (Mani, Utsunomiya, Japan) was inserted into the oval window and the surrounding bone was resected, and then electrical cautery (Solid State Electrosurgery, MS-1500, MERA, Senko Medical Instrument Manufacturing Co., Ltd., Tokyo, Japan) was applied through the file. Penicillin G potassium (3000 U/kg, Meiji Seika Pharma, Tokyo, Japan) and buprenorphine (3 μg/kg, Lepetan, Otsuka, Tokyo, Japan) were administered subcutaneously prior to returning the animals to their cages. The success of the vestibular lesion (VL) was confirmed by observing the swimming behavior of all the mice in warm water. Mice with complete VL could not identify the direction to reach the water surface [25]. Mice were kept in the aluminum cages for two weeks and then 2*g* hypergravity was applied for 14 days.

### Statistical analysis

The mRNA expressions were analyzed using a one-way analysis of variance (ANOVA), followed by a post hoc test (Fisher’s protected least significant difference test) or student t-test, using SPSS (SPSS Japan Inc.). Significance was set at p < 0.05. All data are presented as the mean ± S.E.M except for S1 Table.

## Results

We investigated the effects of hypergravity (2*g* for 14 days) on the mRNA expression of BDNF and 5-HTRs with special reference to 5-HT1AR, 5-HT1BR and 5-HT2AR, because these receptors are involved in various physiological and pathological functions in the brain. We also investigated mRNA expression three days after the exposure to hypergravity in order to examine neural plasticity to recover from hypergravity-induced changes.

### Effects of gravity changes on the mRNA expression of BDNF

**Fig 1:**
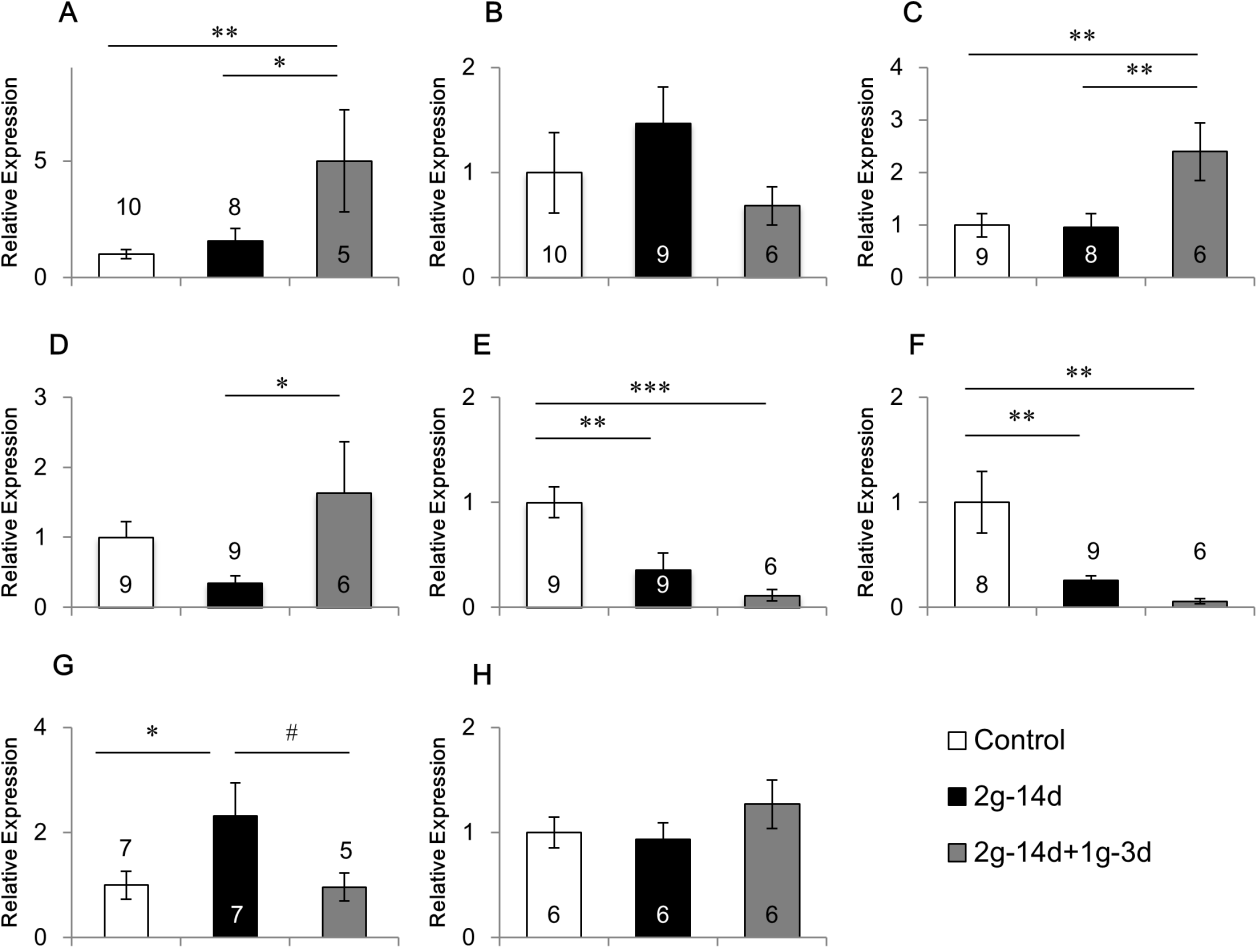
Effects of gravity changes on the mRNA expression of BDNF and TrkB. The mRNA expression of BDNF in the medial prefrontal cortex (A), caudate putamen (B), amygdala (C), dorsal hippocampus (D), ventral hippocampus (E), hypothalamus (F), and cerebellum (G), and TrkB in the hypothalamus (H). 2*g*-14d:14-day exposure to 2*g*, 2*g*-14d+1*g*-3d:3-day recovery after 14-day exposure to 2*g*. 0.05≦ # p<0.07, * p<0.05, ** p<0.01, *** p<0.001.

Gravity changes affected BDNF expression in various brain regions (Fig 1). ANOVA revealed that a main group effect was significant in the BDNF mRNA expression in the medial prefrontal cortex (F(2,20) = 4.880, p<0.05; Fig 1A), amygdala (F(2,20) = 5.449, p<0.05; Fig 1C), ventral hippocampus (F(2,21) = 9.937, p<0.01; Fig 1E), hypothalamus (F(2,20) = 7.282, p<0.01; Fig 1F), and tended to be significant in the dorsal hippocampus (F(2,21) = 3.115, p = 0.065; Fig 1D) and cerebellum (F(2,16) = 2.930), p = 0.082; Fig 1G). The mRNA expression of BDNF was decreased in the ventral hippocampus (p<0.01) and hypothalamus (p<0.01) after 2*g* hypergravity exposure, whereas increased in the cerebellum (p<0.05). The decrease of BDNF expression in the ventral hippocampus and hypothalamus was maintained for three days after returning from 2*g* hypergravity to 1*g* normal gravity (p<0.001 and p<0.01, respectively, as compared with controls). In contrast, 2*g* hypergravity had no significant effects on BDNF expression in the medial prefrontal cortex, caudate putamen, amygdala and dorsal hippocampus. Interestingly, three days after hypergravity was returned to normal gravity, BDNF expression was increased in the medial prefrontal cortex (p<0.05; Fig 1A), amygdala (p<0.01; Fig 1C) and dorsal hippocampus (p<0.05; Fig 1D). These results suggest that the decelerating gravity change from 2*g* to 1*g*, and not accelerating gravity from 1*g* to 2*g* or 2*g* itself may be effective for the modulation of BDNF expression in these brain regions.

Although the mRNA expression of BDNF was decreased by hypergravity in the hypothalamus, its receptor TrkB was not affected by gravity changes (Fig 1H).

### Effects of gravity changes on the mRNA expression of 5-HTRs

**Fig 2:**
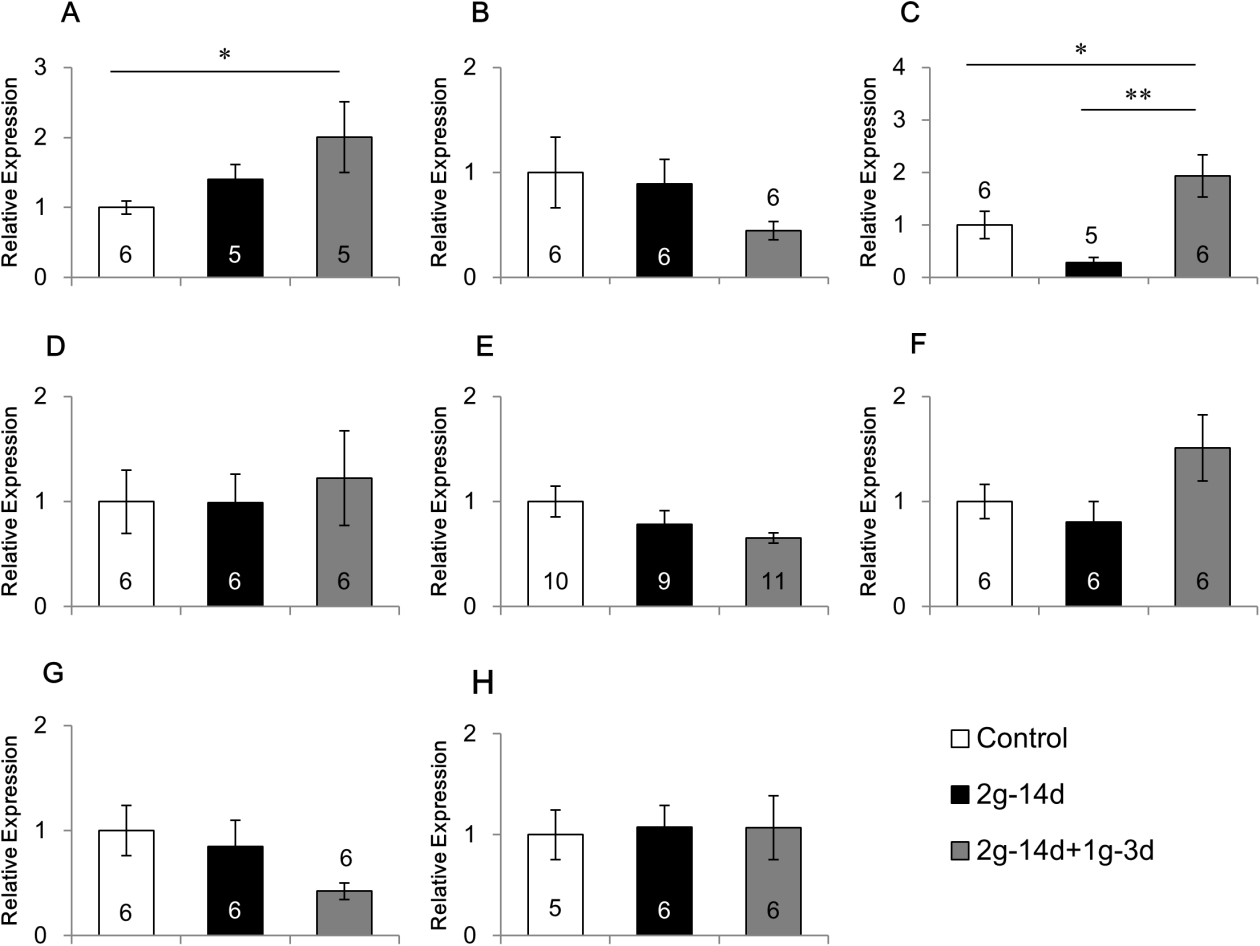
Effects of gravity changes on the mRNA expression of 5-HT1AR. The mRNA expression of 5-HT1A receptor in the medial prefrontal cortex (A), caudate putamen (B), amygdala (C), dorsal hippocampus (D), ventral hippocampus (E), hypothalamus (F), dorsal raphe (G) and cerebellum (H). 2*g*-14d:14-day exposure to 2*g*, 2*g*-14d+1*g*-3d:3-day recovery after 14-day exposure to 2*g*. * p<0.05, ** p<0.01.

**Fig 3:**
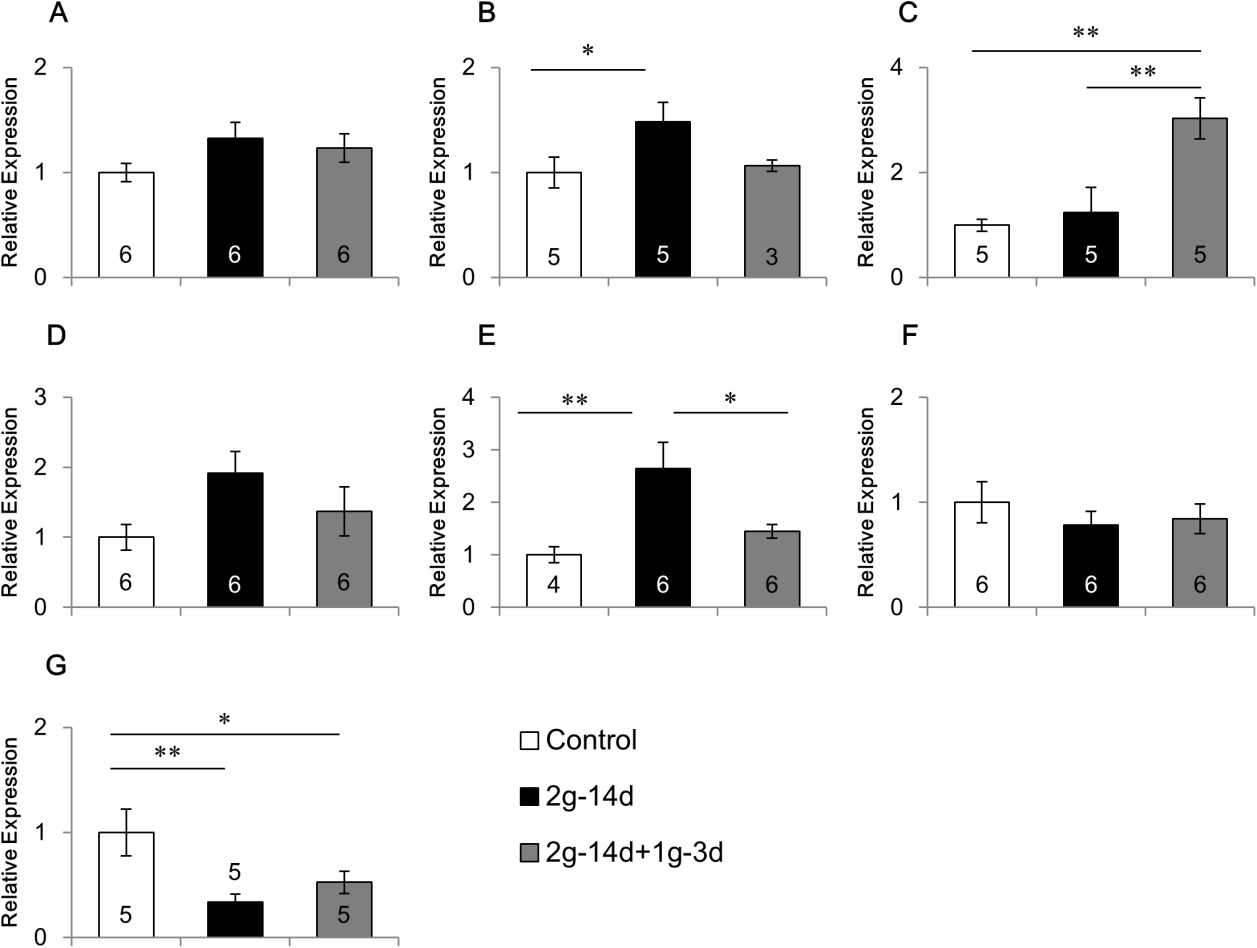
Effects of gravity changes on the mRNA expression of 5-HT1BR. The mRNA expression of 5-HT1B receptor in the medial prefrontal cortex (A), caudate putamen (B), amygdala (C), dorsal hippocampus (D), ventral hippocampus (E), hypothalamus (F), and cerebellum (G). 2*g*-14d:14-day exposure to 2*g*, 2*g*-14d+1*g*-3d:3-day recovery after 14-day exposure to 2*g*. * p<0.05, ** p<0.01.

**Fig 4:**
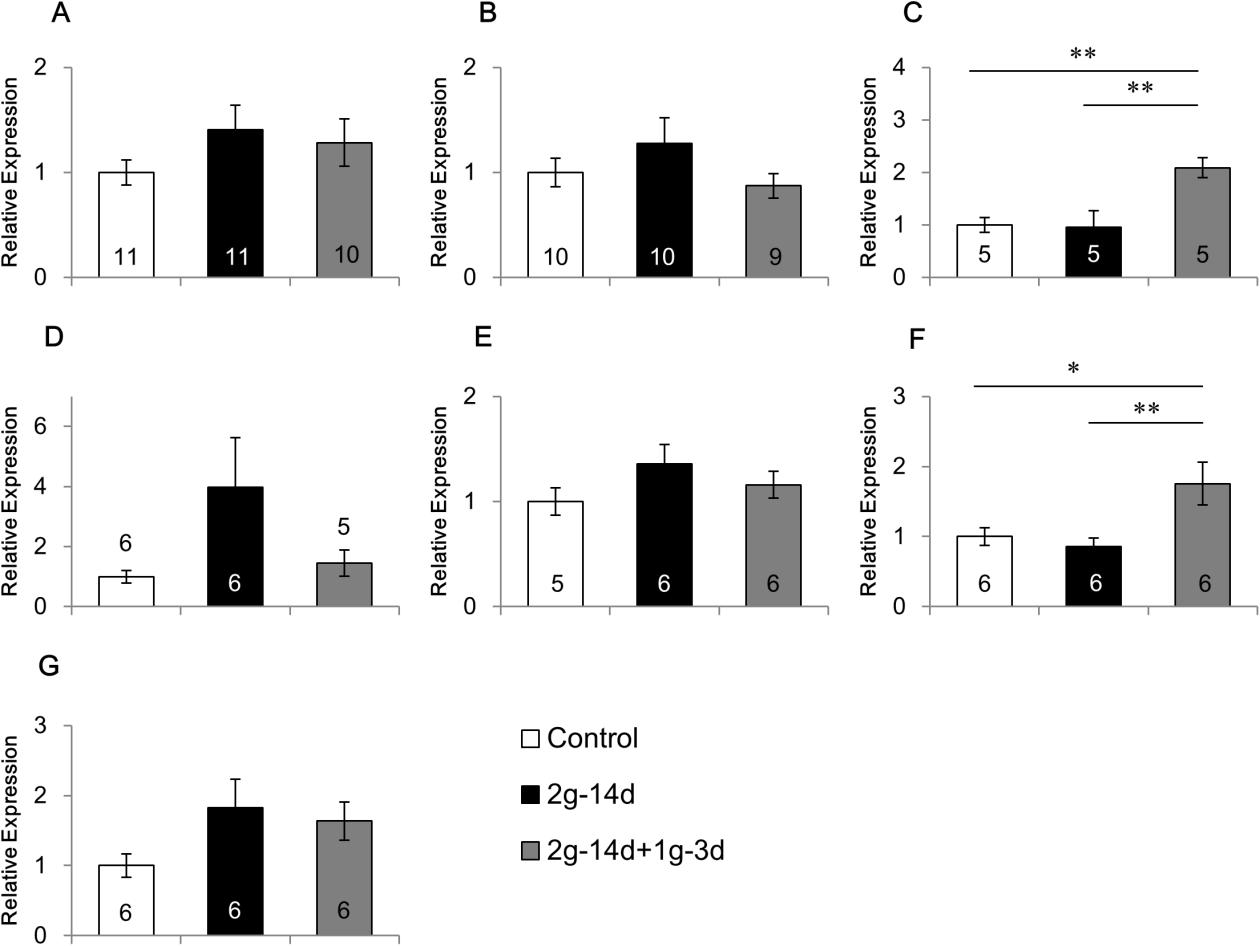
Effects of gravity changes the mRNA expression of 5-HT2AR. The mRNA expression of 5-HT2A receptor in the medial prefrontal cortex (A), caudate putamen (B), amygdala (C), dorsal hippocampus (D), ventral hippocampus (E), hypothalamus (F), and cerebellum (G). 2*g*-14d:14-day exposure to 2*g*, 2*g*-14d+1*g*-3d:3-day recovery after 14-day exposure to 2*g*. * p<0.05, ** p<0.01.

Gravity changes showed differential effects on the mRNA expression of 5-HTRs, depending on the receptor subtypes and regions of the brain (Figs 2–4, S2 Fig).

First, we examined the effects of gravity changes on the mRNA expression of 5-HT1AR (Fig 2). ANOVA revealed that a main group effect was significant in the amygdala (F(2,14) = 7.430, p<0.01; Fig 2C), and tended to be significant in the medial prefrontal cortex (F(2,13) = 2.928, p = 0.089; Fig 2A). No significant group effects were observed in other brain regions. In the amygdala, hypergravity did not change 5-HT1AR mRNA expression. However, three days after returning from hypergravity to normal gravity, 5-HT1AR mRNA expression was increased as compared with the control mice (p<0.05) and the mice exposed to hypergravity (p<0.01). In the medial prefrontal cortex, three days after returning from hypergravity to normal gravity, 5-HT1AR mRNA expression was increased as compared with the control mice (p<0.05).

Next, we examined the effects of gravity changes on 5-HT1BR mRNA expression (Fig 3). ANOVA revealed that a main group effect was significant in the amygdala (F(2,12) = 9.403, P<0.01; Fig 3C), ventral hippocampus (F(2,13) = 6.023, p<0.05 Fig 3E) and cerebellum (F(2,12) = 5.232, p<0.05; Fig 3G), and tended to be significant in the caudate putamen (F(2,10) = 2.957, p = 0.098; Fig 3B). The expression of 5-HT1BR mRNA was increased in the caudate putamen (p<0.05) and ventral hippocampus (p<0.01), but decreased in the cerebellum (p<0.01) by hypergravity exposure. The increase in the ventral hippocampus was recovered during the three-day interval after returning to normal gravity (p<0.05), but the decrease in the cerebellum was not recovered. In the amygdala, three days after returning from hypergravity to normal gravity, 5-HT1BR mRNA expression was increased as compared with the control animals (p<0.01) and the mice exposed to hypergravity (p<0.01), although the hypergravity did not change the mRNA expression.

Fig 4 shows the effects of gravity changes on the expression of 5-HT2AR mRNA. ANOVA revealed that a main group effect was significant in the amygdala (F(2,12) = 8.016, p<0.01; Fig 4C) and hypothalamus (F(2,15) = 5.721, p<0.05; Fig 4F), and no significant group effects were observed in other brain regions. In the amygdala and hypothalamus, no change was observed after hypergravity. However, three days after returning from hypergravity to normal gravity, 5-HT2AR mRNA expression was increased in the amygdala as compared with the control animals (p<0.01) and the mice exposed to hypergravity (p<0.01), and in the hypothalamus as compared with the control animals (p<0.05) and the mice exposed to hypergravity (p<0.01).

Finally, we examined the effects of gravity changes on the mRNA expression of several 5-HTRs and Tph2, the 5-HT synthesizing enzyme (S2 Fig). ANOVA revealed no significant group effect in the gravity changes on the mRNA expression of 5-HT2CR and 5-HT7R in the dorsal hippocampus (S2 Fig), 5-HT4R and 5-HT7R in the ventral hippocampus (S2 Fig). In addition, gravity changes did not affect the mRNA expression of Tph2 in the dorsal raphe (S2 Fig).

### Effects of vestibular organ lesions on the gravity change-induced alteration of gene expression

**Fig 5:**
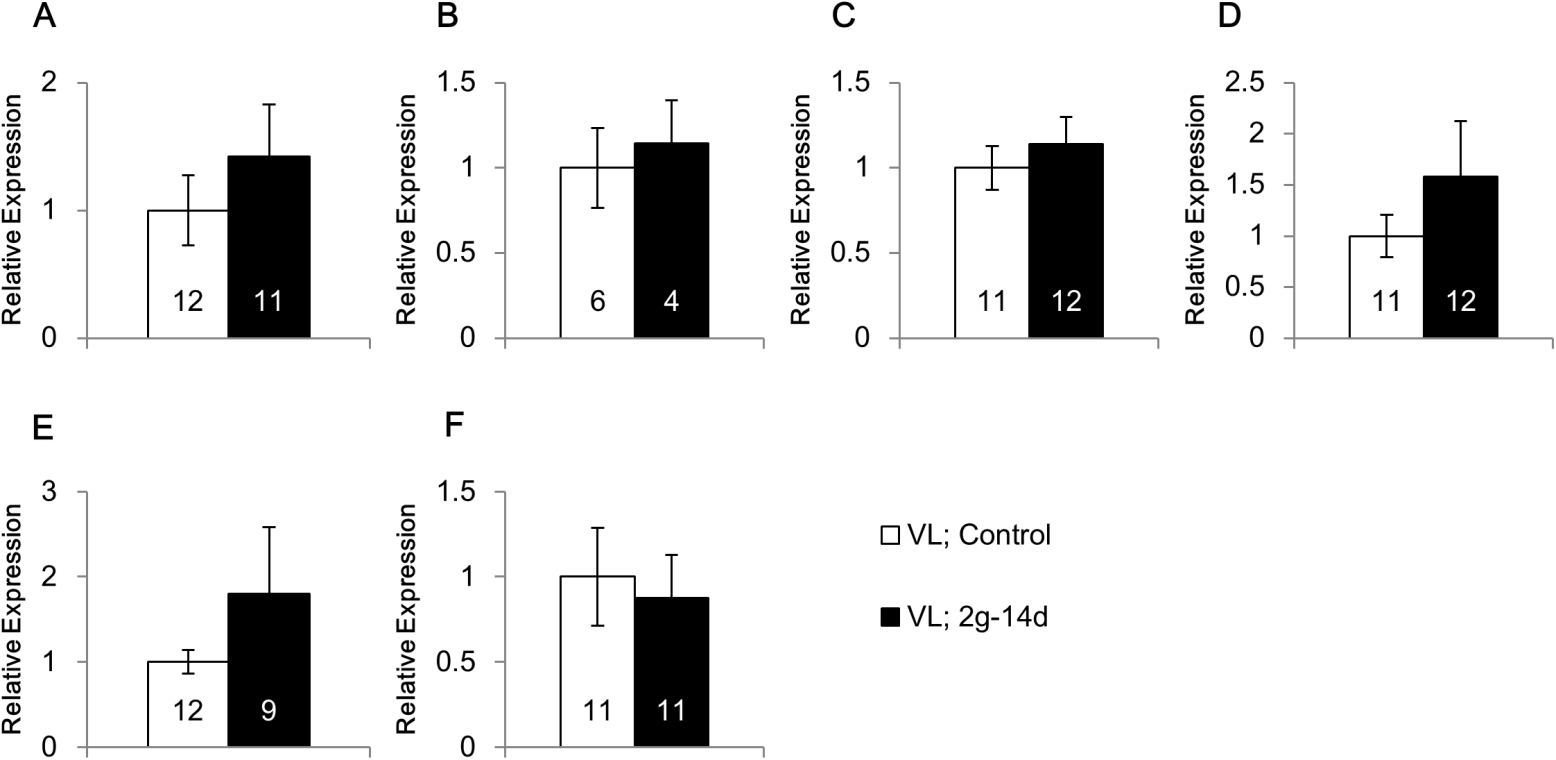
Effects of gravity change on the mRNA expression in the vestibular lesioned mice. The mRNA expression of BDNF in the ventral hippocampus (A), hypothalamus (B) and cerebellum (C), and that of 5-HT1B receptor in the caudate putamen (D), ventral hippocampus (E) and cerebellum (F). VL; Control: Vestibular lesioned mice under normal gravity, VL; 2*g*-14d: Vestibular lesioned mice exposed to 2*g* for 14 days.

To investigate the roles of vestibular organ in the modulation of gene expression under hypergravity, we made surgical lesions of the vestibular organ. As was shown above, the mRNA expression of BDNF was decreased by the hypergravity exposure in the ventral hippocampus and hypothalamus, whereas increased in the cerebellum (Fig 1). In addition, the mRNA expression of 5-HT1BR was decreased in the cerebellum, whereas increased in the caudate putamen and ventral hippocampus (Fig 3). After the surgical lesions of the vestibular organ, the significant effects of hypergravity exposure were lost on BDNF expression in the ventral hippocampus (Fig 5A), hypothalamus (Fig 5B) and cerebellum (Fig 5C), and 5-HT1BR expression in the caudate putamen (Fig 5D), ventral hippocampus (Fig 5E) and cerebellum (Fig 5F).

**Fig 6:**
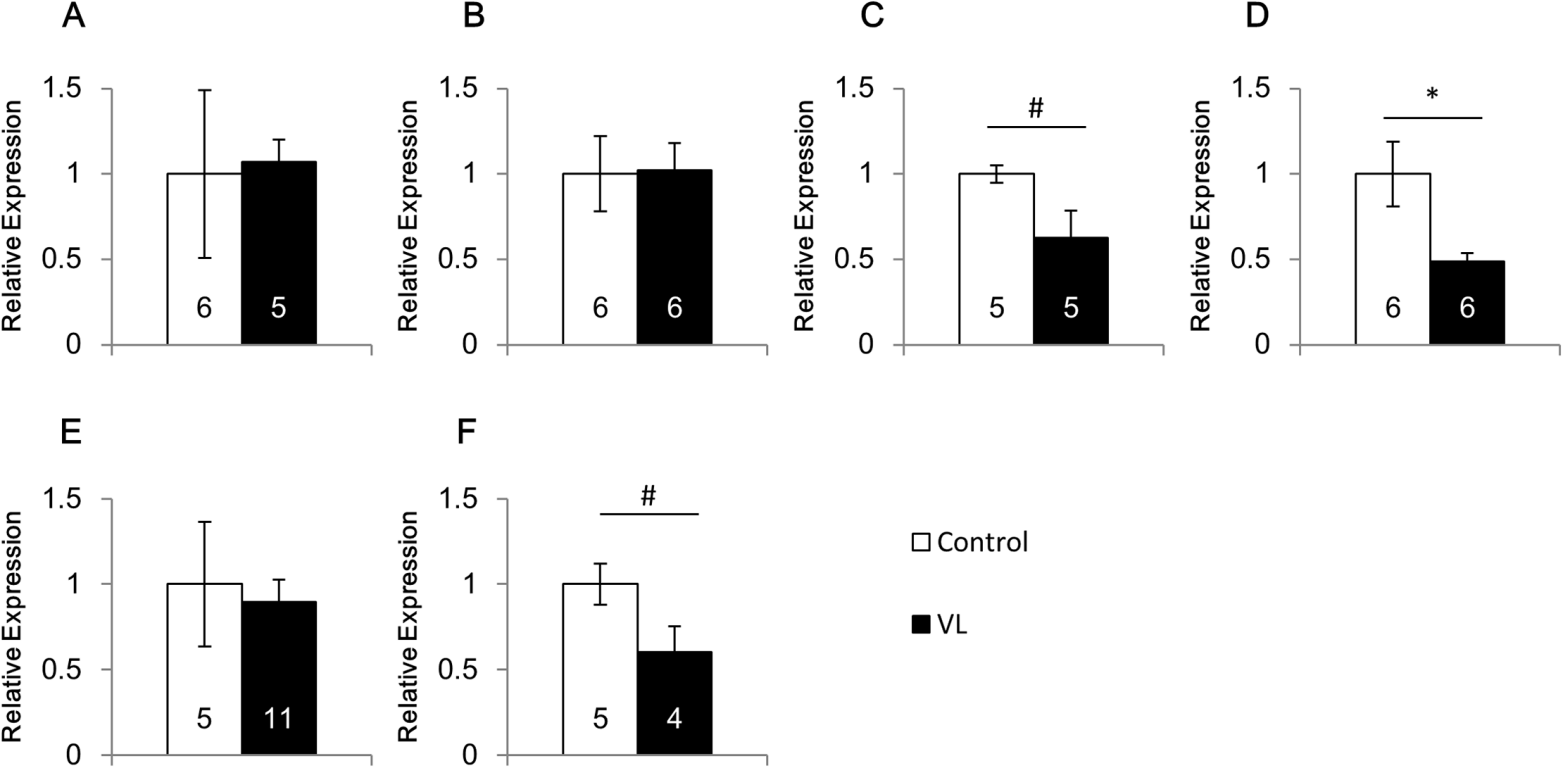
Effects of vestibular lesion on the mRNA expression of BDNF and 5-HT1BR. The mRNA expression of BDNF in the ventral hippocampus (A), hypothalamus (B) and cerebellum (C) and that of 5-HT1B receptor in the caudate putamen (D), ventral hippocampus (E) and cerebellum (F). Control: mice with intact vestibular organ, VL: mice with vestibular lesion. 0.05≦ # p<0.07, * p<0.05.

We next examined the effects of vestibular lesion on the expression of BDNF and 5-HT1BR by comparing vestibular lesioned mice with control (no lesion) mice under normal gravity (Fig 6). The lesion did not show any significant effects on the mRNA expression of BDNF in the ventral hippocampus and hypothalamus (Fig 6A and 6B), and the mRNA expression of 5-HT1BR in the ventral hippocampus (Fig 6E). In contrast, the vestibular lesion tended to decrease the mRNA expression of BDNF (p = 0.053, Fig 6C) and 5-HT1BR (p = 0.068, Fig 6F) in the cerebellum and also decreased the 5-HT1BR mRNA expression significantly in the caudate putamen (p<0.05, Fig 6D).

## Discussion

We investigated the effects of centrifugation-induced gravity changes on gene expression of BDNF and 5-HTRs in the mouse brain. Hypergravity (2*g* for 14 days) and/or the decelerating gravity change from 2*g* to 1*g* modulated gene expression in some regions of the brain. The effects of gravity changes were dependent on gene types and regions of the brain.

### Technical considerations

We examined the effects of hypergravity on gene expression in the brain using the same experimental paradigm as in the previous studies [24,25]. To evaluate the effects of hypergravity, there are several technical points to be considered in the present study. First, to examine the effects of hypergravity, control mice might also be rotated at 1*g* gravity. However, we used the stationary 1*g* control without rotation, because our purpose was to find correlation between the effects of hypergravity on gene expression and those on behaviors shown in the previous studies which used the stationary 1*g* control [7,9,10]. Second, we sampled the brains of mice which were treated with 2*g* hypergravity after returning to 1*g* environment condition. This procedure includes decelerating gravity, and the sampling time after cessation of centrifugation varied from several minutes to hours, which may obscure the effects of hypergravity. To reduce the variation of the after-centrifugation sampling time, we dissected brains by 3 experimenters.

### Effects of gravity changes on gene expression of BDNF and 5-HTRs

In the present study, hypergravity down-regulated BDNF gene expression in the ventral hippocampus and hypothalamus, and up-regulated BDNF gene expression in the cerebellum. These results suggest that the BDNF gene in these brain regions may be more vulnerable to hypergravity than in other regions. In contrast, BDNF gene expression was up-regulated in the medial prefrontal cortex, amygdala and dorsal hippocampus when gravity was returned from 2*g* to 1*g*. Because hypergravity did not change the gene expression, these results suggest that decelerating gravity change but not hypergravity may be effective for modulation of the gene expression. The deceleration of gravity change may be experienced after launch and landing during spaceflight.

Several studies have examined the effects of spaceflight on the gene expression of BDNF and 5-HT receptors in the mouse brain. It was reported that one-month spaceflight did not change BDNF gene expression in the brain regions including the frontal cortex, visual cortex, caudate putamen, hippocampus, hypothalamus and midbrain raphe nuclei [16]. The effects on 5-HT1AR, 5-HT2AR and 5-HT3R were also examined in the same spaceflight, and it was reported that the spaceflight decreased 5-HT2AR gene expression in the hypothalamus without any significant effects on the expression of 5-HT1AR, 5-HT2AR and 5-HT3R in other brain regions [17]. These effects were different from those of the present study. There are several reasons which explain the differences in the effects. First, the effects of microgravity and hypergravity are different. Second, the duration of the gravity change (1 month vs 14 days) is different, and the adaptation to gravity changes may occur during the spaceflight. Third, in the spaceflight, mice were exposed to hypergravity at launch and re-entry in addition to the microgravity, which may reduce the effects of microgravity.

In addition to the microgravity, Del Signore et al. [11] performed a microarray analysis after hypergravity (2*g*, 1 hour daily for 5 consecutive days) using a centrifuge, and reported that many genes were up-regulated while a few genes down-regulated in the hippocampus. However, neither BDNF nor 5-HTRs were changed in the microarray analysis. Considering that the hypergravity affected the expression of BDNF, 5-HT1AR and 5-HT1BR in some brain regions including the hippocampus in the present study, the condition of hypergravity may be milder to induce changes in BDNF and 5-HTRs [11].

A previous study reported opposite responses to environmental factors in BDNF expression between the hippocampus and amygdala [28]. BDNF expression was down-regulated by chronic restraint stress in the hippocampus, but up-regulated in the basolateral amygdala. Concomitantly, both dendrite shrinkage and spine loss were induced by restraint stress in the hippocampus, whereas dendrite growth and increase of spine density were induced in the basolateral amygdala [29,30]. These contrasting structural changes were also reported in the hippocampus and amygdala of patients suffering from major depressive and anxiety disorders [31,32]. The present study showed that hypergravity down-regulated the expression of BNDF mRNA in the ventral hippocampus but had no effect in the amygdala. In contrast, at three days after returning from hypergravity to normal gravity, the ventral hippocampus and amygdala showed opposite response in the BDNF mRNA expression as compared with control mice. It may be interesting to investigate the impact of altering gravity on the structural changes of dendrites and dendritic spines in these brain regions.

### Role of the vestibular organ in the effects of hypergravity on gene expression

Considering that hypergravity elevates the concentration of serum corticosterone and activates neurons in the hypothalamic paraventricular nucleus, a brain center for stress responses, gravity changes can be considered to be a stressor [8,33]. Interestingly, these stress responses were abolished by vestibular lesion, suggesting that they are mediated through the vestibular organ. Information about the gravity is received by the vestibular organ and then transferred to the brain. In addition, hypergravity may stimulate the peripheral body systems such as skin, skeletal system, and muscular system independently of the vestibular organ. To investigate the role of vestibular organ in the mRNA expression in response to gravity changes, we made surgical lesions of the vestibular organ. In intact mice, the mRNA expression of BDNF was affected by the hypergravity in the ventral hippocampus, hypothalamus and cerebellum (Fig 1). In addition, 5-HT1BR mRNA expression was changed in the caudate putamen, ventral hippocampus and cerebellum (Fig 3). After the lesions of the vestibular organ, these effects of hypergravity exposure were lost on the expression of BDNF and 5-HT1BR in these brain regions (Fig 5). These results suggest that information sensing by the vestibular organ may play a crucial role in the modulation of gene expression of BDNF and 5-HT1BR under hypergravity.

To further examine the roles of vestibular organ in the gene expression, we compared the gene expression between mice with lesioned vestibular organ and those with intact vestibular organ (Fig 6). The lesion of the vestibular organ showed differential effects depending on the brain regions. Thus, the lesion did not affect the mRNA expression of BDNF and/or 5-HT1BR in the ventral hippocampus and hypothalamus. However, the lesion decreased the expression of these mRNAs in the cerebellum and caudate putamen. These results showed that the vestibular lesion itself affects the expression of BDNF and 5-HT1BR in the cerebellum and caudate putamen, but has no effect in the ventral hippocampus and hypothalamus. These differences may be derived from the neural connections between the vestibular organ and each brain region. Taken together with the effects of the hypergravity after the vestibular organ lesion, it was suggested that the gene expression of BDNF and 5-HT1BR in the ventral hippocampus and hypothalamus is not affected by the activity of the vestibular organ in normal 1*g* conditions, but the vestibular organ is involved in the hypergravity-induced changes of the gene expression in these brain regions. In contrast, in the cerebellum and caudate putamen, the vestibular organ modulates the gene expression of BDNF and 5-HT1BR in normal 1*g* conditions and hypergravity-induced changes.

There may be interactions in the gravity change-induced expression between BDNF and 5-HT. We showed previously that hypergravity elevates 5-HT concentration in the cerebrospinal fluid, which is completely abolished by the vestibular lesion [34]. In addition, intensive interactions between 5-HT and BDNF in the brain were reported [35]. Taken together, brain 5-HT might play a crucial role in the modulation of BDNF expression via the vestibular organ under gravity changes. Further studies are needed to verify the causal relationship between 5-HT system and BDNF expression.

### Relationship between behavioral changes and gene expression of BDNF and 5-HTRs

Animal experiments on gravity changes have reported that both hypergravity and microgravity have deleterious effects on rodent brain function and behavior. Hypergravity impairs learning ability [6,7,10], and also increases the anxiety level and stress response [5,9]. However, molecular mechanisms responsible for the effects of gravity changes on behavior have not been fully understood.

Among functional molecules, BDNF may be involved in mediating the effects of gravity changes on behavior. BDNF is a neurotrophic factor that regulates brain development and plasticity [36]. In the adult brain, BDNF regulates various brain functions such as cognition, emotion and stress response [19,21,23]. The reduction of BDNF is involved in the pathogenesis of mood disorders such as depression [37–39]. Also reported from the animal experiments was that BDNF expression is decreased in the hippocampus by stress, whereas it is increased in the amygdala [28]. In the present study, hypergravity down-regulated BDNF expression in the ventral hippocampus and hypothalamus, and the deceleration of gravity from 2*g* to 1*g* up-regulated BDNF expression in the medial prefrontal cortex, amygdala and dorsal hippocampus (Fig 1). Considering that these brain regions are involved in mood and anxiety, changes of BDNF expression in these brain regions may mediate the effects of gravity changes on mood and anxiety directly or indirectly. Causal relationship between the changes of BDNF expression and behavior in response to the hypergravity needs to be determined carefully.

In addition to BDNF, the 5-HT system may be also involved in mediating the effects of gravity changes on behavior. We have previously shown that hypergravity increases 5-HT concentration in rodent cerebrospinal fluid [34]. 5-HTRs are widely distributed in the brain, and animal experiments using gene-knockout techniques and pharmacological experiments using receptor agonists/antagonists have reported that multiple 5-HTRs such as 5-HT1AR, 5-HT1BR and 5-HT2AR are involved differentially in emotion and cognition [20,40–43]. The roles of 5-HT1AR are most characterized among these 5-HTRs. Extensive animal experiments indicated that dysfunction of 5-HT1AR induces anxiety and mood disorders. In human patients suffering from a major depressive disorder, the reduction of 5-HT1AR has been revealed in the frontal cortex, hippocampus, amygdala and dorsal raphe by positron emission tomography (PET) studies and post-mortem analyses [44]. In the present study, 5-HT1AR expression was not changed by hypergravity, but modulated by gravity deceleration in the prefrontal cortex and amygdala. The roles of 5-HT1AR expression in various behaviors in response to gravity changes need to be examined.

In the present study, the hypergravity up-regulated 5-HT1BR expression in the caudate putamen and ventral hippocampus while down-regulated in the cerebellum. It was also up-regulated in the amygdala three days after returning from hypergravity to normal gravity. Although the precise roles of 5-HT1BR in neuropsychiatric function remain to be examined, pharmacological studies provided evidence showing that the 5-HT1BR agonist increases anxiety-like behavior and impairs cognition, whereas an antagonist improves cognition [40,45,46]. In addition, expression of 5-HT1BR was up-regulated following exposure to stress in the rodent hippocampus and cortex [47,48]. Taken together, the modulation of 5-HT1BR expression found in the present study may give some insight to explain the brain mechanisms involved in behavioral changes following the alteration of gravity.

In contrast to 5-HT1AR, it was suggested that 5-HT2AR may possess anxiogenic and depressive effects, considering that 5-HT2AR antagonists have anxiolytic and antidepressant actions [49–51]. Consistently, depressed patients often showed increased 5-HT2AR densities, suggesting the involvement of 5-HT2AR overexpression in the pathogenesis of major depressive disorders [52,53]. However, we can not exclude the possibility that the increased 5-HT2AR densities may reflect the lack of 5-HT release in the major depressive disorder. In the present study, hypergravity did not change 5-HT2AR expression in any brain regions. However, the 5-HT2AR expression was up-regulated in the amygdala and hypothalamus three days after returning from hypergravity to normal gravity. These results suggest that 5-HT2AR expression was up-regulated under gravity changes which may induce deleterious effects on behaviors.

Overall, we showed that gravity changes affect gene expression of BDNF and 5-HTRs (5-HT1AR, 5-HT1BR and 5-HT2AR) in some brain regions. These changes may constitute molecular bases that underlie gravity change-induced behavioral alterations. Further studies are needed to examine the causal relationship between changes of gene expression and behavior under gravity changes.

## Supporting information

S1 Fig Analysed brain regions. The photographs of coronal brain slices (left side) and corresponding brain atlas (right side). (A) medial prefrontal cortex, (B) caudate putamen, (C) dorsal hippocampus, (D) hypothalamus, (E) amygdala, (F) ventral hippocampus, (G) dorsal raphe, (H) cerebellum. Areas surrounded by dotted lines showed the analysed brain regions. The brain atlas is from The Mouse Brain in Stereotaxic Coordinates 3rd Edition Franklin & Paxinos.
(TIF)

S2 Fig Effects of gravity changes on the mRNA expression of 5-HT2CR, 5-HT4R, 5-HT7R and Tph2. The mRNA expression of 5-HT2C receptor in the dorsal hippocampus (A), 5-HT4 receptor in the ventral hippocampus (B), 5-HT7 receptor in the dorsal hippocampus (C) and ventral hippocampus (D), and Tph2 in the dorsal raphe (E). 2*g*-14d:14-day exposure to 2*g*, 2*g*-14d+1*g*-3d:3-day recovery after 14-day exposure to 2*g*.
(TIF)

S1 Table The ratio of the weight of soleus muscle to the body weight. We repeated similar experiments twice (Exp.1 and 2). 2*g*-14d:14-day exposure to 2*g*, 2*g*-14d+1*g*-3d:3-day recovery after 14-day exposure to 2*g*. n = 6 for the number of mice in each group. mean ± SD *p <0.05 vs control.
(XLSX)

S2 Table The mRNA expression of 18S rRNA under gravity changes. There were no significant differences in the mRNA expression of 18S rRNA under different gravity changes. “n” shows the number of mice in each group. mean ± S.E.M.
(XLSX)

## References

[1] Leone G. The effect of gravity on human recognition of disoriented objects. Brain Research Reviews. 1998; 28: 203–214. 9795218 10.1016/s0165-0173(98)00040-x

[2] Manzey D, Lorenz B. Mental performance during short-term and long-term space flight. Brain Research Reviews. 1998; 28: 215–221. 9795225 10.1016/s0165-0173(98)00041-1

[3] Reschke MF, Bloomberg JJ, Harm DL, Paloski WH, Layne C, McDonald V. Posture, locomotion, spatial orientation, and motion sickness as a function of space flight. Brain Research Reviews. 1998; 28: 102–117. 9795167 10.1016/s0165-0173(98)00031-9

[4] Grabherr L, Mast FW. Effects of microgravity on cognition: The case of mental imagery. Journal of Vestibular Research. 2010; 20: 53–60. doi: 10.3233/VES-2010-0364 20555167 10.3233/VES-2010-0364

[5] Santucci D, Francia N, Aloe L, Alleva E. Neurobehavioural responses to hypergravity environment in the CD-1 mouse. Journal of gravitational physiology. 2002; 9: 39–40. 14703677

[6] Mandillo S, Del Signore A, Paggi P, Francia N, Santucci D, Mele A, et al Effects of acute and repeated daily exposure to hypergravity on spatial learning in mice. Neuroscience Letters. 2003; 336: 147–150. doi: 10.1016/S0304-3940(02)01282-X 12505614 10.1016/s0304-3940(02)01282-x

[7] Mitani K, Horii A, Kubo T. Impaired spatial learning after hypergravity exposure in rats. Cognitive Brain Research. 2004; 22: 94–100. doi: 10.1016/j.cogbrainres.2004.08.002 15561505 10.1016/j.cogbrainres.2004.08.002

[8] Petrak J, Mravec B, Jurani M, Baranovska M, Tillinger A, Hapala I, et al Hypergravity-induced increase in plasma catecholamine and corticosterone levels in telemetrically collected blood of rats during centrifugation. Annals of the New York Academy of Sciences. 2008; 1148: 201–208. doi: 10.1196/annals.1410.060 19120110 10.1196/annals.1410.060

[9] Gue´guinou N, Bojados M, Jamon M, Derradji H, Baatout S, Tschirhart E, et al Stress response and humoral immune system alterations related to chronic hypergravity in mice. Psychoneuroendocrinology. 2012; 37: 137–147. doi: 10.1016/j.psyneuen.2011.05.015 21724335 10.1016/j.psyneuen.2011.05.015

[10] Bojados M, Jamon M. The long-term consequences of the exposure to increasing gravity levels on the muscular, vestibular and cognitive functions in adult mice. Behavioural Brain Research. 2014; 264: 64–73. doi: 10.1016/j.bbr.2014.01.018 24509308 10.1016/j.bbr.2014.01.018

[11] Del Signore A, Mandillo S, Rizzo A, Di Mauro E, Mele A, Negri R, et al Hippocampal gene expression is modulated by hypergravity. European Journal of Neuroscience. 2004; 19: 667–677. doi: 10.1111/j.1460-9568.2004.03171.x 14984417 10.1111/j.0953-816x.2004.03171.x

[12] Ishii M, Tomizawa K, Matsushita M, Matsui H. Exposure of mouse to high gravitation forces induces long-term potentiation in the hippocampus. Acta Medica Okayama. 2004; 58: 143–149. 15471436 10.18926/AMO/32113

[13] Frigeri A, Iacobas DA, Iacobas S, Nicchia GP, Desaphy JF, Camerino DC, et al Effect of microgravity on gene expression in mouse brain. Experimental Brain Research. 2008; 191: 289–300. doi: 10.1007/s00221-008-1523-5 18704384 10.1007/s00221-008-1523-5 PMC2651838

[14] Sarkar P, Sarkar S, Ramesh V, Kim H, Barnes S, Kulkarni A, et al Proteomic analysis of mouse hypothalamus under simulated microgravity. Neurochemical Research. 2008; 33: 2335–2341. doi: 10.1007/s11064-008-9738-1 18473167 10.1007/s11064-008-9738-1 PMC2740374

[15] Santucci D, Kawano F, Ohira T, Terada M, Nakai N, Francia N, et al Evaluation of gene, protein and neurotrophin expression in the brain of mice exposed to space environment for 91 Days. PLoS ONE. 2012; 7: e40112 doi: 10.1371/journal.pone.0040112 22808101 10.1371/journal.pone.0040112 PMC3392276

[16] Naumenko VS, Kulikov AV, Kondaurova EM, Tsybko AS, Kulikova EA, Krasnov IB, et al Effect of actual long-term spaceflight on BDNF, TrkB, p75, Bax and Bcl-xl genes expression in mouse brain regions. Neuroscience. 2015; 284: 730–736. doi: 10.1016/j.neuroscience.2014.10.045 25451288 10.1016/j.neuroscience.2014.10.045

[17] Popova NK, Kulikov AV, Kondaurova EM, Tsybko AS, Kulikova EA, Krasnov IB, et al Risk neurogenes for long-term spaceflight: dopamine and serotonin brain system. Molecular Neurobiology. 2015; 51: 1443–1451. doi: 10.1007/s12035-014-8821-7 25084757 10.1007/s12035-014-8821-7

[18] Tsybko AS, Ilchibaeva TV, Kulikov AV, Kulikova EA, Krasnov IB, Sychev VN, et al Effect of microgravity on glial cell line-derived neurotrophic factor and cerebral dopamine neurotrophic factor gene expression in the mouse brain. Journal of Neuroscience Research. 2015; 93: 1399–1404. doi: 10.1002/jnr.23600 25944479 10.1002/jnr.23600

[19] Martinowich K, Manji H, Lu B. New insights into BDNF function in depression and anxiety. Nature Neuroscience. 2007; 10: 1089–1093. doi: 10.1038/nn1971 17726474 10.1038/nn1971

[20] Pytliak M, Vargova V, Mechírová V, Felšöci M. Serotonin receptors—from molecular biology to clinical applications. Physiological Research. 2011; 60: 15–25. 20945968 10.33549/physiolres.931903

[21] Gray JD, Milner TA, Mcewen BS. Dynamic plasticity: The role of glucocorticoids, brain-derived neurotrophic factor and other trophic factors. Neuroscience. 2013; 239: 214–227. doi: 10.1016/j.neuroscience.2012.08.034 22922121 10.1016/j.neuroscience.2012.08.034 PMC3743657

[22] Dayer A. Serotonin-related pathways and developmental plasticity: relevance for psychiatric disorders. Dialogues in Clinical Neuroscience. 2014; 16: 29–41. 24733969 10.31887/DCNS.2014.16.1/adayer PMC3984889

[23] Notaras M, Hill R, van den Buuse M. A role for the BDNF gene Val66Met polymorphism in schizophrenia? A comprehensive review. Neuroscience and Biobehavioral Reviews. 2015; 51: 15–30. doi: 10.1016/j.neubiorev.2014.12.016 25562189 10.1016/j.neubiorev.2014.12.016

[24] Morita H, Obata K, Abe C, Shiba D, Shirakawa M, Kudo T, et al Feasibility of a short-arm centrifuge for mouse hypergravity experiments. PLoS ONE. 2015; 10: e0133981 doi: 10.1371/journal.pone.0133981 26221724 10.1371/journal.pone.0133981 PMC4519191

[25] Tateishi R, Akiyama N, Miyauchi M, Yoshinaga R, Sasanuma H, Kudo T, et al Hypergravity provokes a temporary reduction in CD4+CD8+ thymocyte number and a persistent decrease in medullary thymic epithelial cell frequency in mice. PLoS ONE. 2015; 10: e0141650 doi: 10.1371/journal.pone.0141650 26513242 10.1371/journal.pone.0141650 PMC4626100

[26] Akatsu S, Ishikawa C, Takemura K, Ohtani A, Shiga T. Effects of prenatal stress and neonatal handling on anxiety, spatial learning and serotonergic system of male offspring mice. Neuroscience Research. 2015; 101: 15–23. doi: 10.1016/j.neures.2015.07.002 26163770 10.1016/j.neures.2015.07.002

[27] Abe C, Iwata C, Shiina T, Shimizu Y, Morita H. Effect of daily linear acceleration training on the hypergravity-induced vomiting response in house musk shrew (Suncus murinus). Neuroscience Letters. 2011; 502:138–142. doi: 10.1016/j.neulet.2011.06.041 21741451 10.1016/j.neulet.2011.06.041

[28] Lakshminarasimhan H, Chattarji S. Stress leads to contrasting effects on the levels of brain derived neurotrophic factor in the hippocampus and amygdala. PLoS One. 2012; 7: e30481 doi: 10.1371/journal.pone.0030481 22272355 10.1371/journal.pone.0030481 PMC3260293

[29] Watanabe Y, Gould E, McEwen BS. Stress induces atrophy of apical dendrites of hippocampal CA3 pyramidal neurons. Brain Research. 1992; 588: 341–345. 1393587 10.1016/0006-8993(92)91597-8

[30] Vyas A, Mitra R, Shankaranarayana Rao BS, Chattarji S, et al Chronic stress induces contrasting patterns of dendritic remodeling in hippocampal and amygdaloid neurons. The Journal of Neuroscience. 2002; 22: 6810–6818. DOI: 20026655 12151561 10.1523/JNEUROSCI.22-15-06810.2002 PMC6758130

[31] Drevets WC, Price JL, Furey ML. Brain structural and functional abnormalities in mood disorders: implications for neurocircuitry models of depression. Brain Structure Function. 2008; 213: 93–118. doi: 10.1007/s00429-008-0189-x 18704495 10.1007/s00429-008-0189-x PMC2522333

[32] Licznerski P, Duman RS. Remodeling of axo-spinous synapses in the pathophysiology and treatment of depression. Neuroscience. 2013; 251: 33–50. doi: 10.1016/j.neuroscience.2012.09.057 23036622 10.1016/j.neuroscience.2012.09.057 PMC3566360

[33] Matsuda T, Gotoh TM, Tanaka K, Gao S, Morita H. Vestibulosympathetic reflex mediates the pressor response to hypergravity in conscious rats: contribution of the diencephalon. Brain Research. 2004; 1028: 140–147. doi: 10.1016/j.brainres.2004.09.004 15527739 10.1016/j.brainres.2004.09.004

[34] Abe C, Tanaka K, Iwata C, Morita H. Vestibular-mediated increase in central serotonin plays an important role in hypergravity-induced hypophagia in rats. Journal of applied physiology (1985). 2010; 109: 1635–1643. doi: 10.1152/japplphysiol.00515.2010 20847126 10.1152/japplphysiol.00515.2010

[35] Martinowich K, Lu B. Interaction between BDNF and serotonin: role in mood disorders. Neuropsychopharmacology. 2008; 33:73–83. doi: 10.1038/sj.npp.1301571 17882234 10.1038/sj.npp.1301571

[36] Barnabe-Heider F, Miller FD. Endogenously produced neurotrophins regulate survival and differentiation of cortical progenitors via distinct signaling pathways. The Journal of Neuroscience. 2003; 23: 5149–5160. 12832539 10.1523/JNEUROSCI.23-12-05149.2003 PMC6741181

[37] Pittenger C, Duman RS. Stress, depression, and neuroplasticity: A convergence of mechanisms. Neuropsychopharmacology. 2008; 33: 88–109. doi: 10.1038/sj.npp.1301574 17851537 10.1038/sj.npp.1301574

[38] Schmidt HD, Banasr M, Duman RS. Future antidepressant targets: Neurotrophic factors and related signaling cascades. Drug Discov Today Ther Strateg. 2008; 5: 151–156. doi: 10.1016/j.ddstr.2008.10.003 19802372 10.1016/j.ddstr.2008.10.003 PMC2739451

[39] Carlino D, De Vanna M, Tongio E. Is Altered BDNF biosynthesis a general feature in patients with cognitive dysfunctions? Neuroscientist. 2013; 19: 345–353. doi: 10.1177/1073858412469444 23242909 10.1177/1073858412469444

[40] Sari Y. Serotonin1B receptors: from protein to physiological function and behavior. Neuroscience and Biobehavioral Reviews. 2004; 28: 565–582. doi: 10.1016/j.neubiorev.2004.08.008 15527863 10.1016/j.neubiorev.2004.08.008

[41] Carr GV, Lucki I. The role of serotonin receptor subtypes in treating depression: a review of animal studies. Psychopharmacology (Berl). 2011; 213:265–287. doi: 10.1007/s00213-010-2097-z 21107537 10.1007/s00213-010-2097-z PMC3374933

[42] Artigas F. Serotonin receptors involved in antidepressant effects. Pharmacology & Therapeutics. 2013; 137: 119–131. doi: 10.1016/j.pharmthera.2012.09.006 23022360 10.1016/j.pharmthera.2012.09.006

[43] Fakhoury M. Revisiting the serotonin hypothesis: implications for major depressive disorders. Molecular Neurobiology. 2015; doi: 10.1007/s12035-015-9152-z 25823514 10.1007/s12035-015-9152-z

[44] Savitz J, Lucki I, Drevets WC. 5-HT1A receptor function in major depressive disorder. See comment in PubMed Commons belowProgress in Neurobiology. 2009; 88: 17–31. doi: 10.1016/j.pneurobio.2009.01.009 19428959 10.1016/j.pneurobio.2009.01.009 PMC2736801

[45] Lin D, Parsons LH. Anxiogenic-like effect of serotonin1B receptor stimulation in the rat elevated plus-maze. Pharmacology Biochemistry and Behavior. 2002; 71: 581–587. 11888549 10.1016/s0091-3057(01)00712-2

[46] Eriksson TM, Madjid N, Elvander-Tottie E, Stiedl O, Svenningsson P, Ogren SO. Blockade of 5-HT1B receptors facilitates contextual aversive learning in mice by disinhibition of cholinergic and glutamatergic neurotransmission. Neuropharmacology. 2008; 54: 1041–1050. doi: 10.1016/j.neuropharm.2008.02.007 18394658 10.1016/j.neuropharm.2008.02.007

[47] Edwards E, Harkins K, Wright G, Henn FA. 5-HT1b receptors in an animal model of depression. Neuropharmacology. 1991; 30: 101–105. 2046876 10.1016/0028-3908(91)90050-l

[48] Clark MS, Neumaire JF. The 5-HT1B receptor: behavioral implications. Psychopharmacology Bulletin. 2001; 35: 170–185. 12397864

[49] Bressa GM, Marini S, Gregori S. Serotonin S2 receptors blockade and generalized anxiety disorders. A double-blind study on ritanserin and lorazepam. International Journal of Clinical Pharmacology Research. 1987; 7: 111–119. 3108171

[50] Patel JG, Bartoszyk GD, Edwards E, Ashby CR Jr. The Highly selective 5-Hydroxytryptamine (5-HT)2A receptor antagonist, EMD 281014, significantly increases swimming and decreases immobility in male congenital learned helpless rats in the forced swim test. Synapse. 2004; 52: 73–75. doi: 10.1002/syn.10308 14755634 10.1002/syn.10308

[51] Zhang G, Stackman RW Jr. The role of serotonin 5-HT2A receptors in memory and cognition. Frontiers in Pharmacology. 2015; 6: 225 doi: 10.3389/fphar.2015.00225 26500553 10.3389/fphar.2015.00225 PMC4594018

[52] Meyer JH, McMain S, Kennedy SH, Korman L, Brown GM, DaSilva JN, et al Dysfunctional attitudes and 5-HT2 receptors during depression and self-harm. The American Journal of Psychiatry. 2003; 160: 90–99. doi: 10.1176/appi.ajp.160.1.90 12505806 10.1176/appi.ajp.160.1.90

[53] Shelton RC, Sanders-Bush E, Manier DH, Lewis DA. Elevated 5-HT 2A receptors in postmortem prefrontal cortex in major depression is associated with reduced activity of protein kinase A. Neuroscience. 2009; 158: 1406–1415. doi: 10.1016/j.neuroscience.2008.11.036 19111907 10.1016/j.neuroscience.2008.11.036 PMC9208662

