# Supplementary figures and images for: Effects of gravity changes on gene expression of BDNF and serotonin receptors in the mouse brain
Source: PLoS One. 2017 Jun 7;12(6):e0177833. doi: 10.1371/journal.pone.0177833 (PMC5462371; doi:10.1371/journal.pone.0177833)

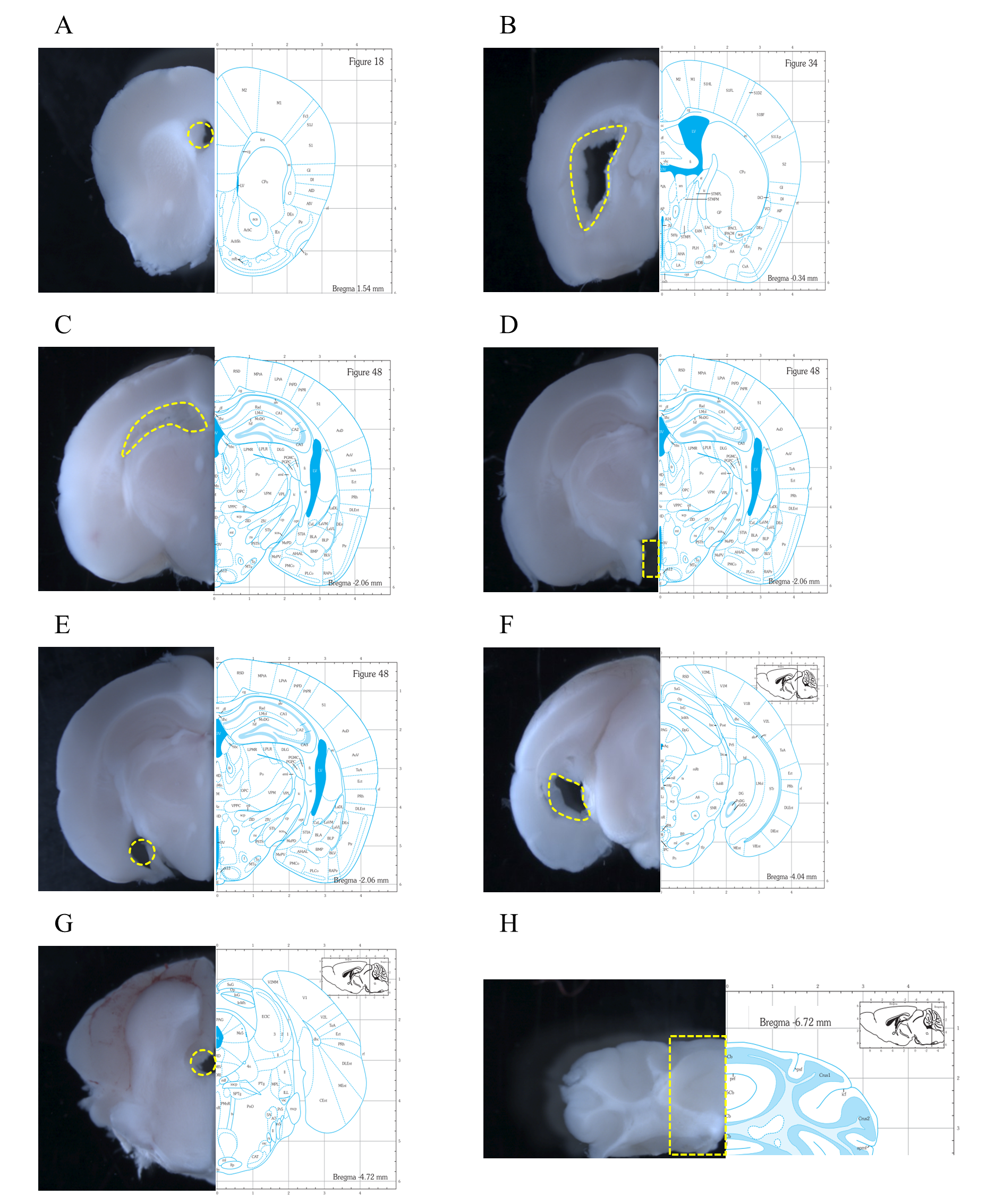

Supplement: S1 Fig — The photographs of coronal brain slices (left side) and corresponding brain atlas (right side). (A) medial prefrontal cortex, (B) caudate putamen, (C) dorsal hippocampus, (D) hypothalamus, (E) amygdala, (F) ventral hippocampus, (G) dorsal raphe, (H) cerebellum. Areas surrounded by dotted lines showed the analysed brain regions. The brain atlas is from The Mouse Brain in Stereotaxic Coordinates 3rd Edition Franklin & Paxinos. (TIF) [file pone.0177833.s001.tif]

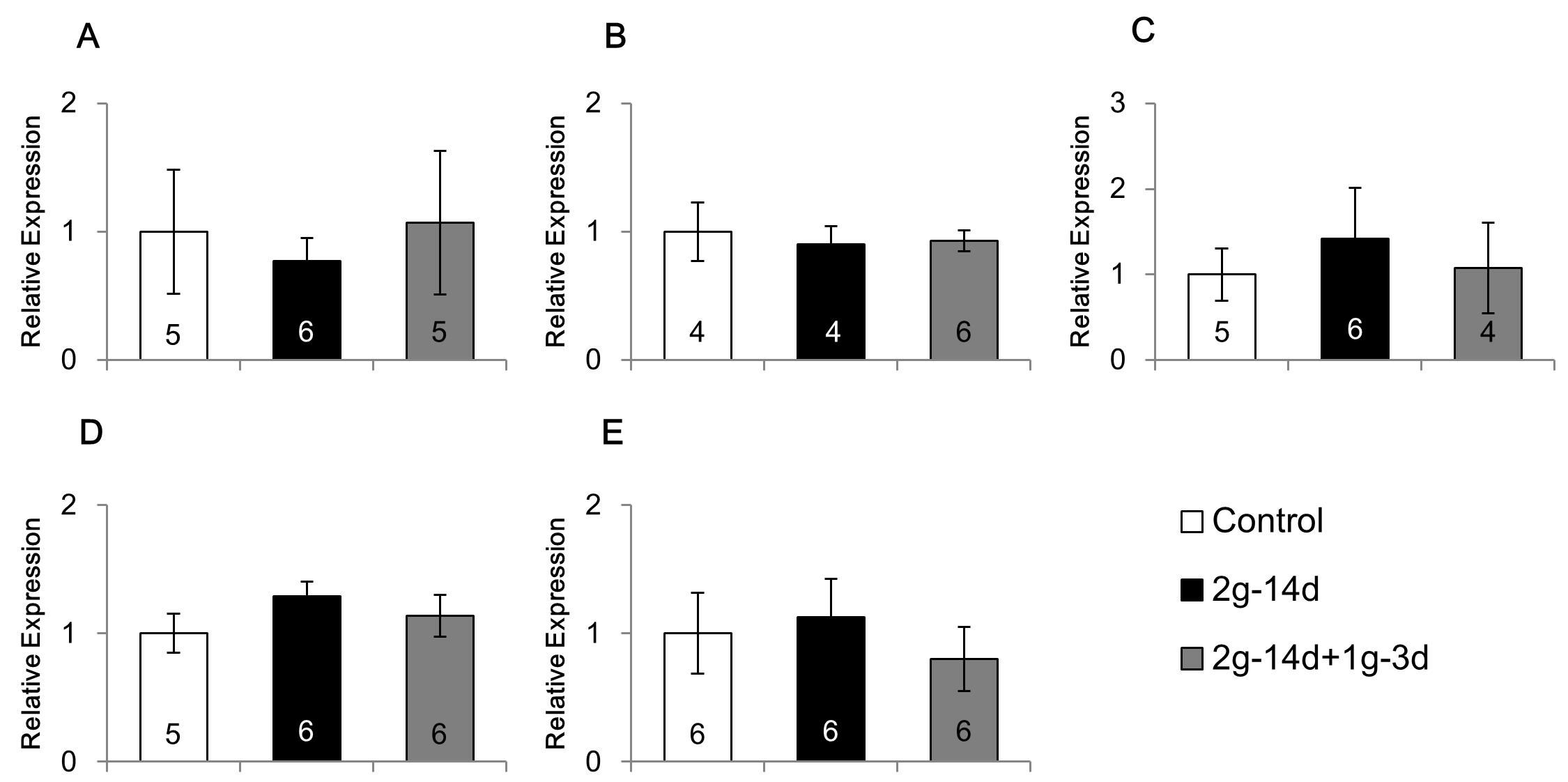

Supplement: S2 Fig — The mRNA expression of 5-HT2C receptor in the dorsal hippocampus (A), 5-HT4 receptor in the ventral hippocampus (B), 5-HT7 receptor in the dorsal hippocampus (C) and ventral hippocampus (D), and Tph2 in the dorsal raphe (E). 2g-14d:14-day exposure to 2g, 2g-14d+1g-3d:3-day recovery after 14-day exposure to 2g. (TIF) [file pone.0177833.s002.tif]
